# Genome-wide association study reveals distinct genetic associations related to leaf hair density in two lineages of wheat-wild relative *Aegilops tauschii*

**DOI:** 10.1038/s41598-022-21713-3

**Published:** 2022-10-19

**Authors:** Mazin Mahjoob Mohamed Mahjoob, Nasrein Mohamed Kamal, Yasir Serag Alnor Gorafi, Hisashi Tsujimoto

**Affiliations:** 1 United Graduate School of Agricultural Sciences, Tottori University, Tottori, 680-8553 Japan; 2 Wheat Research Program, Agricultural Research Corporation, P.O. Box 126, Wad Medani, Sudan; 3 Arid Land Research Center, Tottori University, Tottori, 680-0001 Japan

**Keywords:** Evolution, Genetics, Plant sciences

## Abstract

Wild relatives of modern crops represent a promising source of genetic variation that can be mined for adaptations to climate change. *Aegilops tauschii*, the D-sub-genome progenitor of bread wheat (*Triticum aestivum*), constitutes a reservoir of genetic diversity for improving bread wheat performance and environmental resilience. Leaf hairiness plays an essential biological role in plant defense against biotic and abiotic stress. We investigated the natural variation in leaf hair density (LHD) among 293 *Ae. tauschii* accessions. Genome-wide association studies were performed for LHD with 2430 and 3880 DArTseq derived single nucleotide polymorphism (SNP) markers in two lineages of this species, TauL1 and TauL2, respectively. In TauL1, three marker-trait associations (MTAs) were located on chromosome 2D, whereas in TauL2, eight MTAs were identified, two associations were localized on each of the chromosomes 2D, 3D, 5D, and 7D. The markers explained phenotypic variation (R^2^) from 9 to 13% in TauL1 and 11 to 36% in TauL2. The QTLs identified in chromosomes 2D and 5D might be novel. Our results revealed more rapid and independent evolution of LHD in TauL2 compared to TauL1. The majority of LHD candidate genes identified are associated with biotic and abiotic stress responses. This study highlights the significance of intraspecific diversity of *Ae. tauschii* to enhance cultivated wheat germplasm.

## Introduction

Hairs or trichomes are epidermal protuberances that, depending on species, are located on the aerial parts of plants, such as the leaves, stems, petioles, petals, and seed coat^1^. The presence of leaf trichomes, or leaf hairs (LHs), is common among angiosperms^1^. LHs emerge during the formation of the leaf epidermis and their final density on mature leaves is due to a variety of intercellular interactions^2^. The form and density of LHs differ significantly among plant species, populations, and individuals. LHs are unicellular or multicellular in structure, and they can be straight, spiral, hooked, branched, or unbranched^3,4^. Secondary metabolites (e.g. terpenes and alkaloids) released by glands in certain trichomes can be toxic, repellant, or trap insects and other creatures; these trichomes are also known as glandular trichomes^5^. Certain species develop both glandular and non-glandular leaf trichomes^6,7^.

Several studies have reported on the adaptive importance of LHs in angiosperms. The presence of epidermal hairs acts as a physical barrier against biotic and abiotic stresses on plant surfaces, including herbivory by insects, pathogenic microorganisms, UV light, excessive transpiration, and freezing^8,9^. LHs were shown to be especially important for cultivars that grow in drought conditions^10,11^. Pest damage to cereal leaves is also reduced by non-branched leaf hairs^12–14^.

A previous study in common wheat reported that a dominant gene *Pa* controls the presence or absence of LHs and is located on chromosome 4BS^15^. The density and localization of LHs vary within *T. aestivum* (AABBDD)^16^. Several loci controlling LHs length and density have been reported in wheat. For examples, a single locus for leaf hairiness (*Hl1*) in three different common wheat cultivars was identified on chromosome 4BL based on a monosomic analysis^17^; loci controlling glume pubescence were identified on chromosome 1AS^18–20^; loci regulating leaf hairiness were identified on chromosome 7BS^21^ and chromosome 7D^22^; two loci controlling leaf margin hairiness of the third and fourth leaves were reported on chromosome 4DL; and a distinct locus controlling the hairiness of auricles on the third and fourth leaves was identified on the long arm of chromosome 4B^23^.

*Aegilops tauschii* Coss. (syn. *Ae. squarrosa* L.) is a diploid, self-pollinating goat grass and is the D genome donor of hexaploid wheat^24,25^. *Ae. tauschii* is genetically and morphologically diverse and is classified into the subspecies *tauschii* and *strangulata*^26^. The ssp. *tauschii* has elongated cylindrical spikelets, whereas ssp. *strangulata* has quadrate spikelets and empty glumes^27^. Genomic and chloroplast DNA studies have classified *Ae. tauschii* into three lineages: (1) TauL1, which includes only the ssp. *tauschii*; (2) TauL2, which includes both ssp. *tauschii* and *strangulata*; and (3) TauL3, which is characterized by intermediate spike forms^28–30^. *Ae. tauschii* is expected to have greater LHD than bread wheat because it has a wide geographical range and is adapted to many different harsh environments, including drought and heat conditions, high pathogen burdens, and nutrient-poor soils. Indeed, most *Ae. tauschii* accessions have hair/trichomes on the leaf sheath^28^ as well as hairy auricles^31^. The presence or absence of LHs was studied in *Ae. tauschii* accessions KU-2078 and PI499262 using a mapping population, and a candidate gene was designated as *hfl* on chromosome 3D^28^. In a study by Morihiro and Takumi in 2010^31^, the high-density LH phenotype was observed mainly in Transcaucasus accessions and accessions derived from northern Iran, including four accessions of the *strangulata* subspecies. In contrast, the low-density LH phenotype was mainly found in eastern accessions from Afghanistan and Pakistan. They found that the *Ae. tauschii* germplasm in the eastern habitats was phenotypically distinct from that in the western habitats^31^. Wan et al.^32^ studied the relationship between LH and yield components. They mapped a major QTL for leaf sheath hairiness introgressed from *Ae. tauschii* onto chromosome 4DL in two recombinant inbred lines. They found that a QTL allele resulting in hairy leaf sheaths was significantly and positively associated with increased grain yield and weight per spike.

The study presented here is one of the first to use Genome-Wide Association (GWA) as a powerful tool to detect genetic variants associated with LHD from different lineages in *Ae. tauschii*^33,34^.

The objectives of this study were to (1) assess LHD variation in different *Ae. tauschii* lineages originating from across its natural range, and (2) identify marker-trait associations (MTAs) for LHD in the two main lineages, TauL1 and TauL2. Plant materials used in this study were categorized into TauL1, TauL2, and TauL3 lineages following the previous research^30^. In TauL1, we identified three MTAs on chromosome 2D associated with LHD. In contrast, in TauL2, we identified eight MTAs associated with LHD, two each on chromosomes 2D, 3D, 5D, and 7D. Our results reveal that the TauL1 and TauL2 lineages have distinct loci associated with LHD. The geographic distribution of these alleles is consistent with a model in which the TauL2 lineage evolved faster to adapt to harsh climates.

## Results

### Leaf hair density (LHD) variation in *Ae. tauschii*

**Figure 1:**
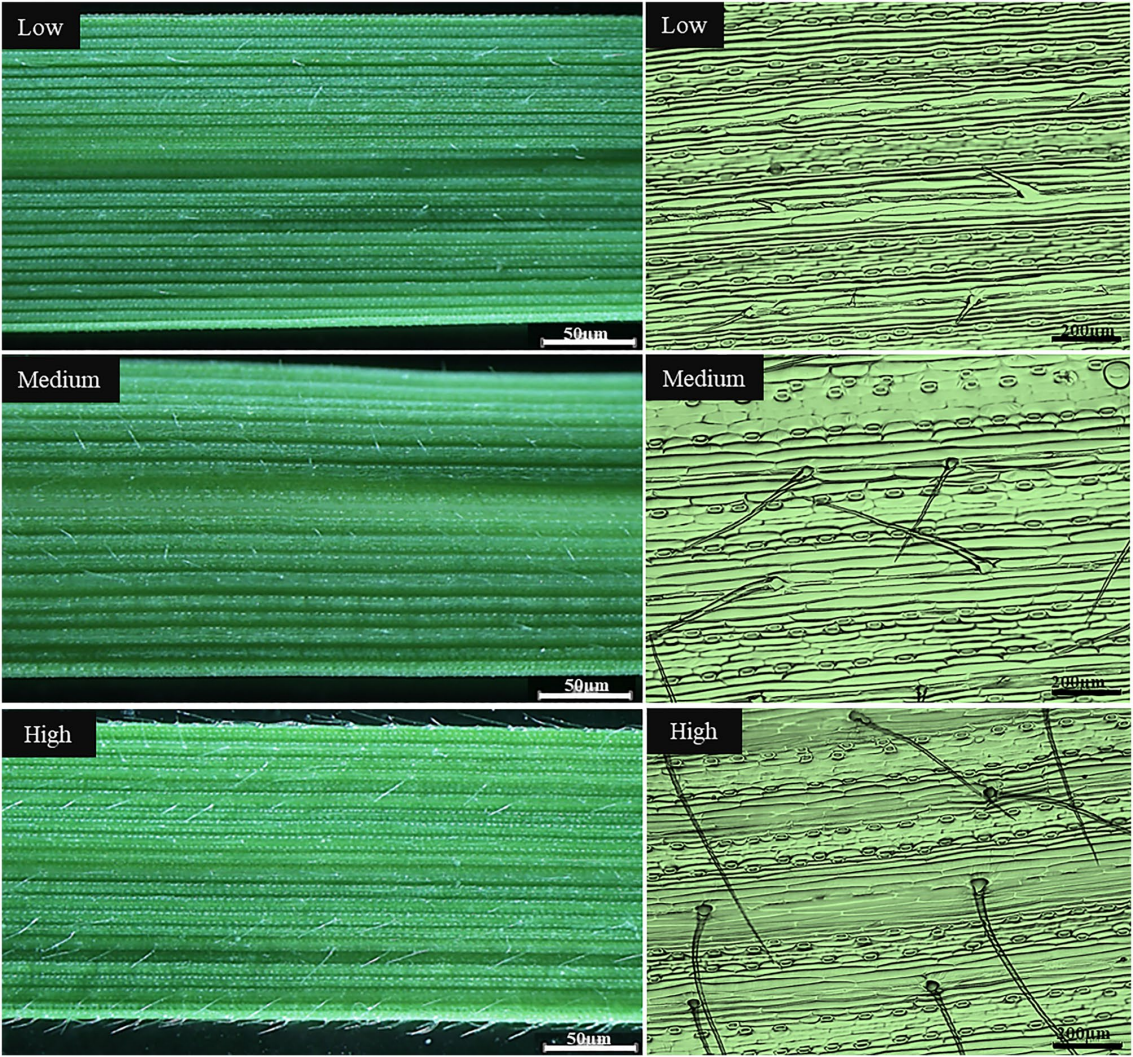
Representative photographs (left side) and scanning microscope photographs (right side) of the leaf adaxial side illustrating the low (1–3), medium (4,5) and high (6,7) leaf density scoring ranges among 293 *Aegilops tauschii* accessions.

To assess LHD variation in *Ae. tauschii*, we used a scoring scale ranging from 1 to 7 using visual inspection of leaf hairs (from the total leaf area): 1 = no visible leaf hairs or less than 10% of leaf pubescence, 2 = visible hairs at approximately 10–15% density, 3 = visible leaf hairs at approximately 15–30% density, 4 = visible leaf hairs at approximately 30–45% density, 5 = visible leaf hairs at approximately 45–55% density, 6 = visible leaf hairs at approximately 55–75% density, and 7 = visible leaf hairs at over > 75% density (Fig. 1). For GWA analyses, we extracted DNA from fresh leaves and accessions were genotyped using the DArTseq platform^30^.

**Figure 2:**
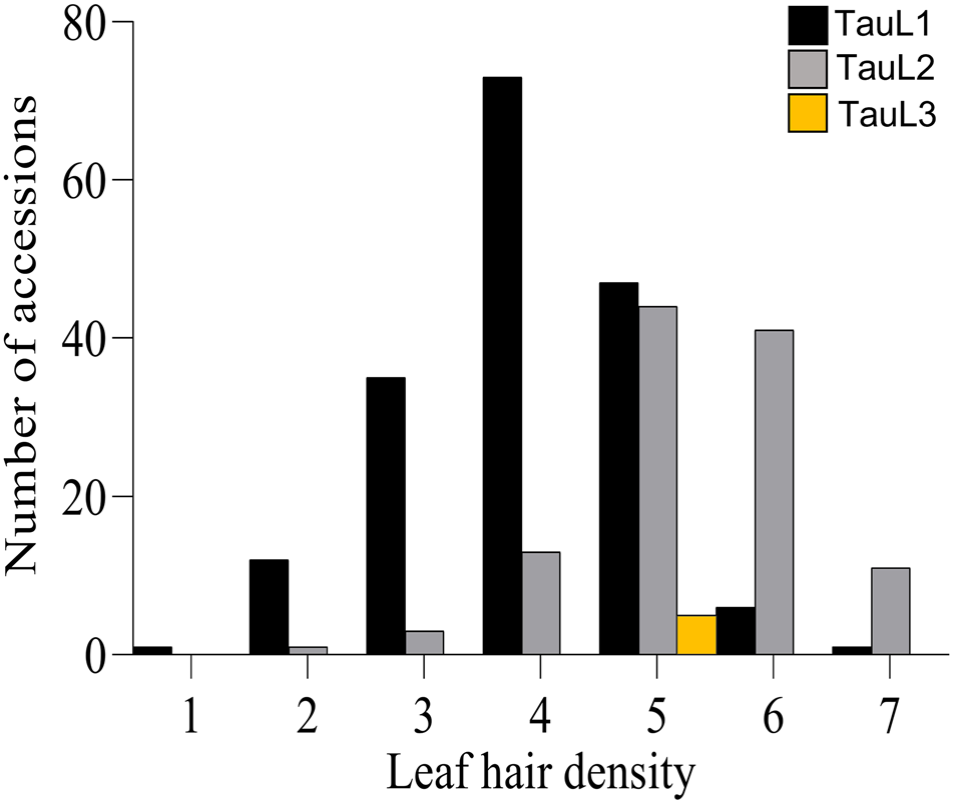
Leaf hair density variation in *Aegilops tauschii* ssp. *tauschii* in TauL1 (175 accessions), TauL2 (113 accessions) and TauL3 (5 accessions) lineages. Black bars represent TauL1, gray bars represent TauL2, and yellow bars represent TauL3.

ANOVA revealed high genetic variation in LHD among accessions (Table 1). In replicate experiments conducted in 2018/2019 and 2019/2020, the effect of genotype × seasonal (G × S) interaction was not significant. LHD variation among accessions was investigated statistically with respect to range, mean, standard deviation, and coefficient of variation. The broad-sense heritability (*H*^2^) for LHD was 0.90. Using the mean LHD calculated between replicate seasons for each accession, we identified one accession with a score of 1, 13 accessions representing 4% of the population with score 2, 38 accessions representing 13% of the population with score 3, 86 accessions representing 29% of the population with score 4, 96 accessions representing 32% of the population with score 5, 47 accessions representing 16% of the population with score 6 and 12 accessions representing 4% of the population with score 7 (Table S1, Fig. 2). These results clearly show that most accessions have LHD ranging from 30 to 55%. After separating *Ae. tauschii* accessions into two lineages, TauL1 and TauL2, we found that these two lineages differed in their LHD: TauL2 accessions have greater LHD than TauL1 accessions (Fig. 2). LHD in 54 TauL1 accessions, representing 30% of the population, exceeded 55%. LHD in 96 TauL2 accessions, representing 84% of the population, exceeded 55%. LHD in all TauL3 accessions ranged from 45 to 55%. These results clearly show that accessions belonging to the TauL2 lineage have higher LHDs than TauL1 and TauL3 (Table S1). Accessions belonging to ssp. *tauschii* exhibited large variation in LHD, ranging from 1% to over 75%, whereas ssp. *strangulata* exhibited a narrower range of phenotypic variation in LHD (45 to over 75%). We found that ssp. *strangulata* (belonging to TauL2) comprised 7 accessions with LHD ranging from 45 to 55%, 7 from 55 to 75%, and 1 with over 75% (Table S1).

**Table 1:** Variance analysis (ANOVA) of leaf hair density (LHD) measured in 293 *Aegilops tauschii* accessions grown in a growth chamber in two seasons (S1: 2018–2019 and S2: 2019–2020).

Season	LHD range	Mean LHD	*p-*value (G)	*p*-value (S)	*p*-value (G × S)	SED± (G)	Heritability (*H*^2^*)*	CV (%)
S1	1.92–6.52	4.21	< 0.001			0.81	0.90	19.3
S2	3.89–5.51	4.76	0.037			1.48		31.2
Mean	1.02–7.32	4.50	< 0.001	< 0.001	1.00	0.73		

SED ± : standard error of differences; CV: coefficient of variation.

Analysis of the relationship between the three lineages in LHD showed no relationship between TauL1 and TauL2, and TauL1 and TauL3 (*r* = 0.0004, 0.0412, respectively), whereas TauL2 and TauL3 were correlated (*r* = 0.21*).

### Genome-wide association studies in *Ae. tauschii*

**Figure 3:**
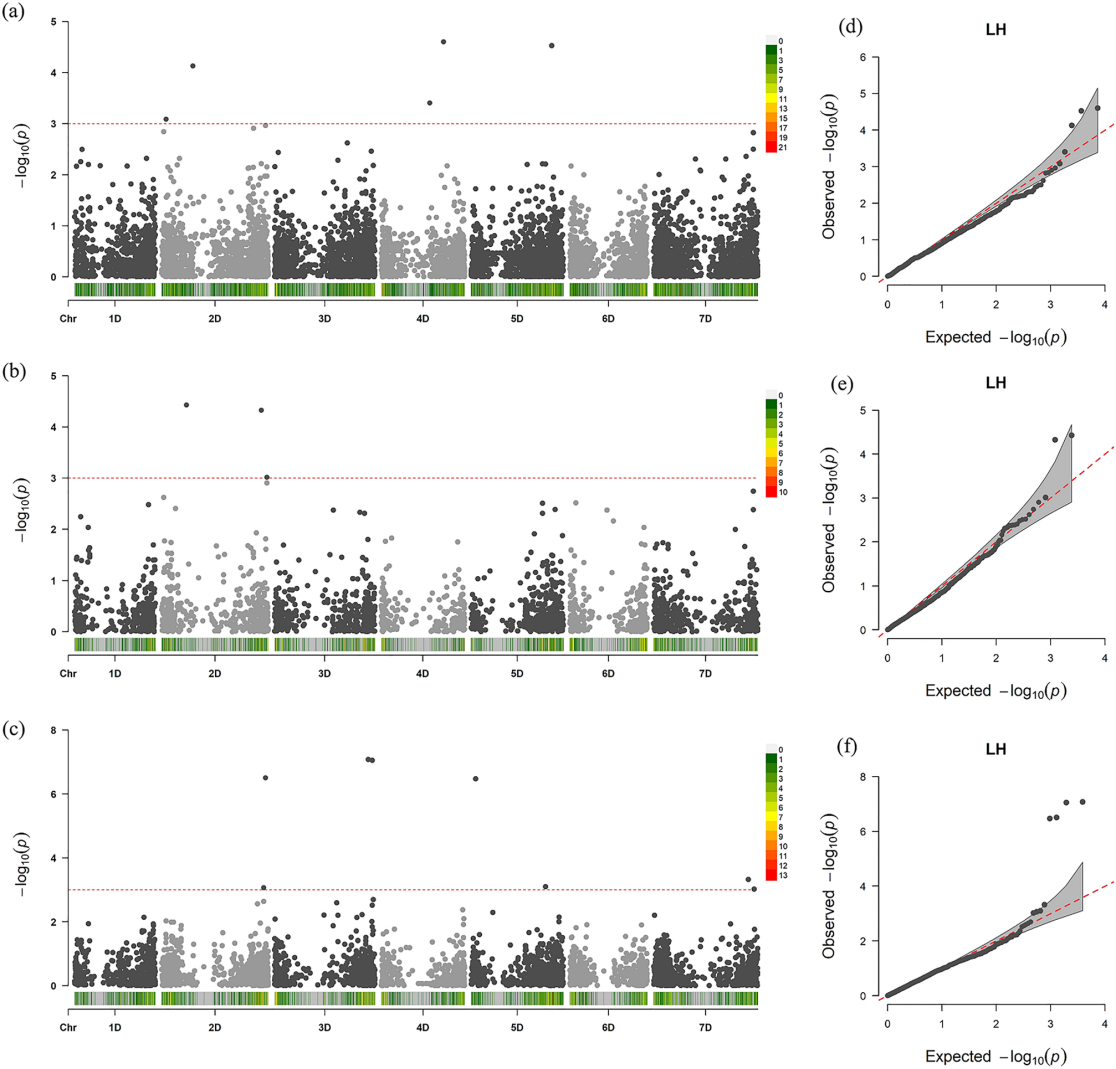
Manhattan plots illustrating *Aegilops tauschii* single nucleotide polymorphism (SNP)-LHD associations detected by a mixed linear model using BLUP values in (**a**) all accessions, (**b**) TauL1, and (**c**) TauL2. Genomic coordinates are displayed along the X-axis, with the negative logarithm of the association p-value for each SNP displayed on the Y-axis. Redline indicates the significance threshold. Q-Q plots showing deviation of GWAS results from the null hypothesis for leaf hairs in (**d**) all accessions, (**e**) TauL1, and (**f**) TauL2.

**Figure 4:**
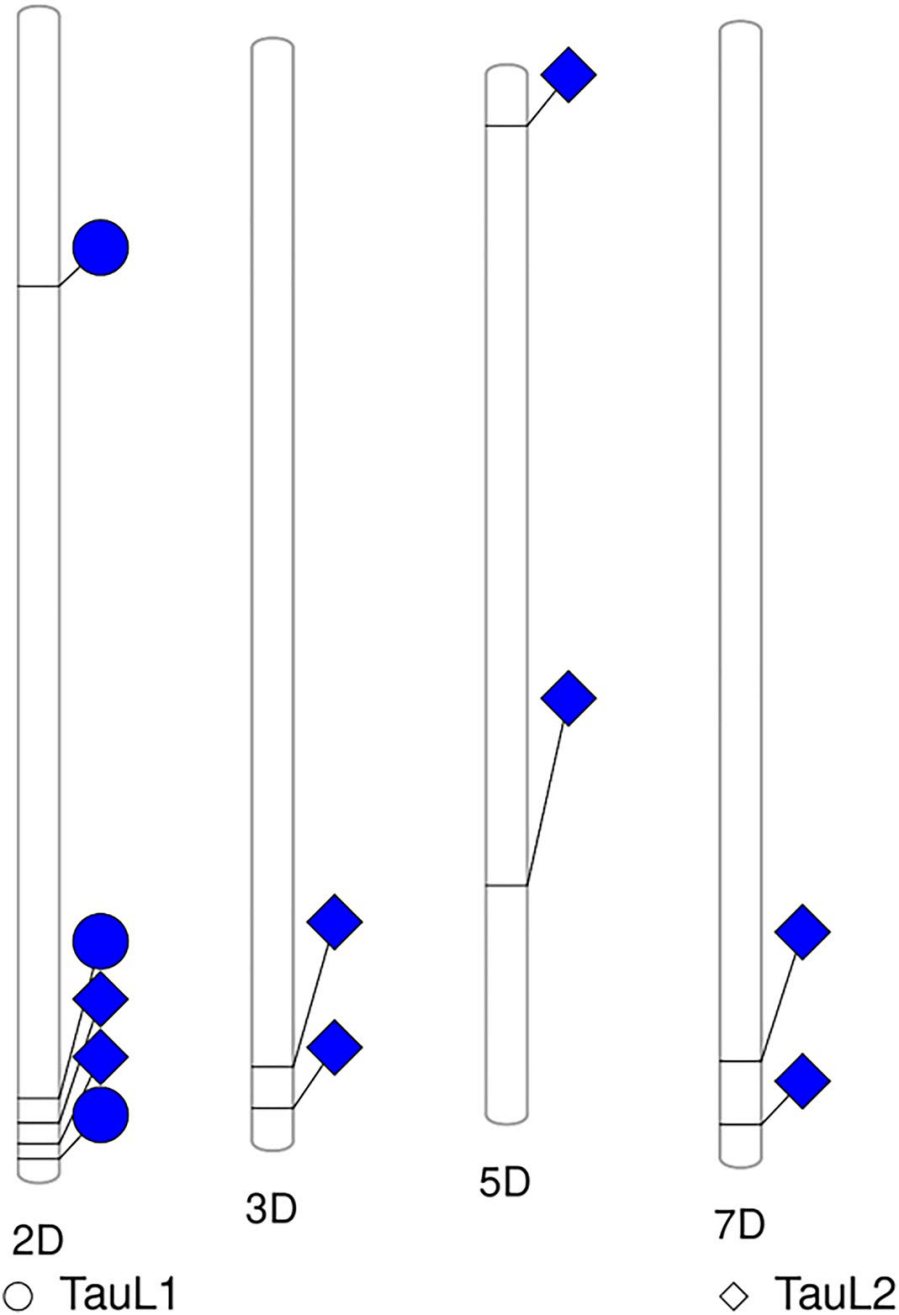
Chromosome phenogram plots of significant LHD-SNP associations detected by GWAS in TauL1 (circles) and TauL2 (diamonds) lineages of *Aegilops tauschii*.

Using the entire population of 293 *Ae. tauschii* accessions, we did not detect any MTAs with high phenotypic effect associated with LHD but detected five minor MTAs. Two minor MTAs were located on each of the chromosomes 2D (at 27.33 and 190.36 Mbp) and 4D (in the range of 297.11—381.74 Mbp) and one on chromosome 5D (at 494.18 Mbp). The contribution of these MTA's to the phenotypic variation (*R*^2^) was negligible and ranged from 5 to 8% (Table 2, Fig. 3a). To identify major MTAs controlling LHD, we independently analyzed the two main lineages (TauL1 and TauL2). In TauL1 accessions, we identified three markers on chromosome 2D associated with LHD: one at 152.02 Mbp and two spanning the region from 609.58 -643.90 Mbp (Table 2, Fig. 3b). In the TauL2 lineage, we detected eight MTAs, two each on chromosomes 2D (in 624.00–635.21 Mbp), 3D (in 573.66–597.67 Mbp), 5D (at 28.93 and 456.68 Mbp), and 7D (in 580.18–616.07 Mbp) (Table 2, Fig. 3c). On chromosome 2D, the two MTAs detected in TauL1 were located in the same region of those identified in TauL2 (Fig. 4). The *R*^2^ values ranged from 9 to 13% in TauL1 and 11 to 36% in TauL2. Thus, GWAS results showed more significant MTAs with a high proportion of phenotypic variance in TauL2 compared to TauL1 (Table 2, Fig. 3).

**Table 2:** Marker-trait associations obtained for all *Aegilops tauschii* accessions, as well as TauL1 and TauL2 lineages analyzed separately.

Accession grouping	Marker	Chromosome	Position	*p*-value	Marker *R*^2^
*All accessions*	32735570|F|0–24	4D	381738725	2.5E−05	0.08
	32736989|F|0–36	5D	494181964	3E−05	0.08
	32752621|F|0–39	2D	190362534	7.4E−05	0.07
	32766324|F|0–32	4D	297111947	0.00039	0.06
	32737380|F|0–17	2D	27328218	0.00082	0.05
TauL1	32763258|F|0–40	2D	152018947	3.7E−05	0.13
	32782271|F|0–48	2D	609577494	4.7E−05	0.13
	32773953|F|0–56	2D	643902850	0.00097	0.09
TaL2	4305083|F|0–54	3D	573661362	8.3E−08	0.36
	32762083|F|0–30	3D	597672424	8.8E−08	0.35
	4269564|F|0–25	2D	635210437	3.1E−07	0.32
	32752435|F|0–29	5D	28933365	3.4E−07	0.32
	32781371|F|0–54	7D	580177726	0.00048	0.13
	32773977|F|0–46	5D	456680160	0.00079	0.14
	32779404|F|0–20	2D	624001770	0.00086	0.12
	32747309|F|0–64	7D	616067185	0.00094	0.11

GWAS used a mixed linear model with markers revealed by DArTseq.

BLAST searches were used to identify candidate genes responsible for variation in LHD in the TauL1 and TauL2 lineages. Lineage-specific markers significantly contributed to the phenotypic variation in LHD, whereas detected markers using all *Ae. tauschii* accessions did not have a significant contribution. Putative candidate genes functioning as LHD regulators in TauL1 and TauL2 are shown in Supplementary Tables S2 and S3. All markers identified in this study are associated with genes playing roles in plant defense against biotic and abiotic stresses (Supplementary Tables S2 and S3). Candidate genes identified in this study had no direct relationship with leaf hair development or similarity to previously discovered LHD regulators.

## Discussion

### Relationships between leaf hair density and geographical distribution

*Ae. tauschii* is widely distributed in Eurasia, ranging from northern Syria and Turkey to western China, and shows a high degree of genetic variation among populations and individuals^35–38^. In this study, LHD in most accessions collected from the western portion was higher than in accessions collected from the east. In other words, LHD was higher in TauL2 than in TauL1 accessions (Table S1, Fig. 2). However, LHD was also high in TauL1 accessions originating from Azerbaijan and Iran. Interestingly, LHDs in TauL3 accessions were intermediate (Table S1, Fig. 2). These results suggest that the geographic variation in *Ae. tauschii* for LHD could be due to the eastward expansion.

Furthermore, the weak correlations between TauL1 and TauL2 in LHD indicate the independent evolution of LHD in these two lineages. The intermediate LHD in TauL3 further supports the previous reports that this lineage might result from hybridization between TauL1 and TauL2.

Due to the distribution of the waxy bloom allele in *Ae. tauschii* populations originating from the southwestern Caspian Sea, Iran, and nearby mountains in Azerbaijan; these populations are believed to be the source of the D genome of bread wheat^39^. Thus, accessions from harsh environments could be a potential source for traits enhancing wheat's biotic and abiotic stress adaptation. Our results clearly show that LHD has spread eastward from the western range of *Ae. tauschii* were consistent with the study of Morihiro and Takumi^31^. On the other hand, the high LHD in TauL1 accessions from Iran and Azerbaijan suggests a relationship between the environment and the increased LHD.

### GWAS of leaf hair density in TauL1 and TauL2

GWAS for LHD was conducted using association mapping panels consisting of 175 accessions belonging to TauL1 and 113 accessions belonging to TauL2^30^. We identified 3 and 8 MTAs in TauL1 and TauL2, respectively (Table 2, Figs. 3, 4). The *R*^2^ values for LHD were higher in TauL2 than in TauL1 (Table 2). We identified four MTAs in TauL2 for which the *R*^2^ was greater than 31%. Furthermore, the *p*-values of the TauL2-specific MTAs were more significant than those obtained for TauL1 or all accessions (Fig. 3d–f). This result is consistent with the lineage-specific differences observed for LHD among accessions.

Several previous studies have reported on LH-related loci in bread wheat. One gene, reported on chromosome 4DL (*QHl.ipk-4D*), was introduced from *Ae. tauschii*^23^. Using the entire *Ae. tauschii* population we found an MTA on chromosome 4D with minor effect that might correspond to the same region identified previously. However, the GWAS in TauL1 and TauL2 did not detect this QTL. This result could be attributed to the low frequency of the associated allele in the independent populations.

This study identified three major MTAs on *Ae. tauschii* chromosome 2D in the TauL1 lineage and eight major MTAs, two each on chromosomes 2D, 3D, 5D, and 7D (*R*^2^ = 11–36%) in the TauL2 lineage. Most of the markers we identified are associated with stress response genes (Supplementary Tables 1 and 2), and we did not find any MTAs previously implicated in regulating LHD.

Interestingly, MTAs on chromosomes 3D, 5D and 7D were specifically detected in TauL2 and not TauL1 (Table 2, Fig. 4). These three QTLs should contribute to the intraspecific variation of the LH morphology in TauL2. The TauL2 accessions possessed higher LHD than the TauL1 accessions, so the TauL2 intraspecific variation could be mainly due to the genetic differences in the 2D QTL regions having the main contribution and the additive effect of the 3D, 5D, and 7D QTLs region that contribute to the subspecies' morphological differentiation. Recently in rice Hamaoka et al. ^40^ reported that the elongation of the macro-hairs through the epidermal cell differentiation is genetically independent of the short macro-hairs on small vascular bundles. In other words, the hair's presence is independent of the density and length, which is also influenced by the environment^40^. Considering our findings and the other reports, the evolution of the LHD in the *Ae. tauschii* lineages can be attributed to the environment prevailing in each lineage distribution range since TauL1 accessions co-exist with TauL2 accessions in the western range have higher LHD than those exist in the eastern part of the lineage range.

Many QTLs for LH traits have been detected on various chromosomes of common wheat^15,17–23,28^, whereas the 2D and 5D QTLs detected in the present study might be novel. QTLs on the 4D and 7D homoeologous loci are already found on common wheat's B genome. However, fine mapping of these QTLs is required to elucidate their association. Furthermore, the production of wheat multiple synthetic derivatives (MSD) will provide an excellent chance to confirm the expression of these QTLs in the bread wheat genome as reported by Gorafi et al. ^41^.

### Importance of *Ae. tauschii* alleles regulating LHD for wheat breeding

Previous research has demonstrated that LHD contributes to resistance and tolerance to a wide range of biotic and abiotic stresses^42^ as well as grain yield and spike weight^32^. Identifying the genetic loci controlling LHD in wheat is a promising approach to improving agronomic performance under biotic and abiotic stresses. Previous studies identified genes underlying LH traits in wheat, barley, and rye. In specific accessions of *Triticum turgidum* var. *dicoccoides*, the hairy leaf sheath trait is determined by complementary genes^43^, but their chromosomal locations are unknown. These genes play essential roles in biotic and abiotic stress responses in wheat*. Ae. tauschii* has been used to create synthetic hexaploid wheat by crossing with tetraploid wheat (*T. turgidum* ssp*. durum*) and subsequent chromosome doubling. Synthetic wheat represents a readily accessible germplasm pool for wheat breeding as it can enhance the variation available in bread wheat^44,45^. Although *Ae. tauschii* accessions have a hairy leaf sheath and common wheat is usually glabrous^15^, LH genes are expressed in the hexaploid genetic background^15^.

LH-related traits in *Ae. tauschii* were transferred to common wheat varieties selected from crosses making use of synthetic hexaploid wheat^46–49^. Our study found that IG 127025, IG 46623, IG 47188, IG 47203, KU-2083, KU-2092, and KU-2093 are accessions with high LHDs. These accessions were also identified as having high LHDs in the study conducted by Morihiro and Takumi^31^. These accessions belong to the TauL2 lineage, which originated from the southwestern Caspian Sea region. With the anticipated pressures of climate change expected to increase environmental stresses on crops, genetic stocks exhibiting high LHD will be useful to widen the narrow genetic base of wheat germplasm to increase resistance to various stresses. Our results clearly show that TauL1 and TauL2 regulate LHD using distinct genes, suggesting an independent evolution in each lineage.

Moreover, as reported by Tsunewaki et al.^50^, the origin of common wheat is restricted to a narrow distribution range within the western habitats of *Ae. tauschii,* and the D-genome donor accessions putatively belong to TauL2 or undiscovered populations^51^. Thus, the 2D and 5D QTLs might not have been integrated into the common wheat genome^28,52^.

Recently, Zhou et al.^53^ developed a platform to introduce genetic variations from 278 *Ae. tauschii* accessions into wheat by combining speed breeding and high-throughput genotyping and phenotyping. Their results provide valuable resources for new gene discovery, genotyping, wheat improvement, and resource utilization. On the other hand, Kumar et al.^54^ established a k-mer-based association mapping pipeline on a diverse panel of 242 *Ae. tauschii* accessions and identified a QTL for LH on chromosome 4DL that corresponds to the QTL identified by Dobrovolskaya et al.^23^. They reported that an uncharacterized *Ae. tauschii* lineage contributed to the initial gene flow into domesticated wheat and understanding the evolution of bread wheat will facilitate the discovery of useful genetic variation from *Ae. tauschii*. Although all these studies provide valuable information using powerful genomic techniques, understanding the wheat evolution should address the evolution of *Ae. tauschii* belongs to different lineages. This study and our previous studies (Mahjoob et al.^30,36^) investigated the traits and genomic regions associated with lineages differences for more efficient utilization of the tremendous resources of *Ae. tauschii* in wheat breeding to biotic and abiotic stress tolerance.

## Conclusion

We studied the phenotypic and genotypic variation in LHD in two lineages of *Ae. tauschii* (TauL1 and TauL2). The results indicated that LHD evolved independently in the two lineages, which is associated with the environment in each lineage range. The two MTAs in TauL2 (2D and 5D) identified in the present study are reported for the first time and might not be presented in the current bread wheat. These QTLs can be used in wheat breeding through direct and indirect crossing. This study highlights the significance of intraspecific diversity and lineage differences of *Ae. tauschii* that should be considered to enhance cultivated wheat germplasm.

## Materials and methods

### Plant materials

To study LHD variation in a wild relative of modern hexaploid wheat a collection of 293 *Ae. tauschii* accessions were used (Supplementary Table S4). AE accessions were received from the Leibniz Institute of Plant Genetics and Crop Plant Research (IPK), Germany; AT accessions from the Faculty of Agriculture, Okayama University, Japan; CGN accessions from the Instituut Voor Planten Veredeling, Landbouwhoge School, Wageningen, the Netherlands; IG accessions from the International Center for Agricultural Research in the Dry Areas (ICARDA), Syria; KU accessions from the Germplasm Institute, Faculty of Agriculture, Kyoto University, Japan; and PI accessions from the US Department of Agriculture. These accessions were sampled from the entire natural species range, from northern Syria and Turkey to western China. In this panel, 175 accessions belonged to TauL1, 113 to TauL2, and 5 to the TauL3 lineage. Of 293 accessions, 278 were the ssp. *tauschii* and 15 were the ssp. *strangulata*^30^.

### Phenotypic evaluation and statistical analysis

Experiments using 293 *Ae. tauschii* accessions were carried out in a growth chamber at the Arid Land Research Center, Tottori University, in 2018/2019 and replicated in 2019/2020. In both years, the growth chamber day/night temperatures were 22/14 °C, with a day length of 12 h, and relative humidity ranged from 60 to 80%.

The experiment was conducted using an augmented complete block design with five blocks. We randomly selected three accessions as checks and replicated them in the five blocks. Five plants of each accession were grown in polyethylene pots (7.2 × 9.0 cm diameter, Tokai Co., Japan).

We evaluated the leaf hair density (LHD) on the first fully expanded leaf. The population was classified into seven clear LHD classes with seven LHD scales. About 15–20 random accessions were selected from each class to confirm the similarities within and among the different class accessions (by counting leaf hair/area using ImageJ software) using the scanning microscope (Hitachi Co., Ltd, Japan). After confirming the scale's suitability to classify all accessions, we visually classified the entire population in both seasons using the seven-point scale by comparing the accessions with the reference accessions in each scale class identified through microscope scanning.

### Statistical analysis

Analysis of the leaf hair density data, including mean, standard deviation, and analysis of variance (F and p-values in one-way ANOVA) for genotypic effect (G) in each season (S) and their interaction (G × S) effects were calculated using Plant Breeding Tools (PBTools) version 1.4 software (International Rice Research Institute, http://bbi.irri.org/products). The accessions were considered as fixed effects and replications as random effects. To estimate the broad sense heritability and calculate the predicted means, the genotypic effect was treated as a random effect in PBTools software.

### DNA extraction, DArTseq genotyping, and genomic analysis

Genomic DNA was extracted using the CTAB method^55^, and DNA samples of (50 μl; 50–100 ng μl^−1^) were sent to Diversity Arrays Technology (DArT) Pty., Ltd, Australia (http://www.diversityarrays.com) for a whole-genome scan using the DArTseq platform. At the DArT facility, DNA of each accession was treated with a combination of restriction enzymes for complexity reduction to obtain a subset of restriction fragments for each accession. The restriction fragments were then sequenced and after quality control aligned to the D genome of wheat_ChineseSpring10 reference genome to identify physical chromosome positions of each SNPs markers^56^. DArTseq generated a total of 42,801 SNP markers from the 293 accessions. Using a filter selection of < 7% missing data, 16,382 SNP markers remained. A SNPs with a minor allele frequency less than 0.05 were removed, and GWA analyses were performed on subsets of *Ae. tauschii*. A total of 7294, 2430, and 3880 SNPs were used for the full set of accessions (including all of the TauL1, TauL2, and TauL3), only TauL1 accessions, and only TauL2 accessions, respectively. We performed GWAS using the LHD predicted mean value for each accession in TASSEL version 5 software^57^. We used a mixed linear model (MLM) with principal component analysis (PCA) and a kinship matrix to account for population structure and cryptic relationships.

Because the Bonferroni-Holm correction for multiple testing (α = 0.05) was too stringent, markers with an adjusted –log_10_ (*p*-value) ≥ 3.0 were regarded as significant. Mixed linear model results were used to generate Manhattan plots and chromosome density plots in R package version 1.2.5033 using the CMplot package version 3.6.2. To associate significant polymorphisms with candidate genes, we performed a BLAST search of the sequences flanking each significant marker against the Chinese Spring RefSeq v. 1.0 wheat reference genome (IWGSC, 2021). The genomic position with the best match was extended by 0.5 Mb in both directions and used in a subsequent BLAST search of the Ensembl *Triticum aestivum* database to find predicted genes or proteins within this region.

### Ethics statement

The authors confirm that the handling of the plant materials used in the study complies with relevant institutional, national, and international guidelines and legislation.

## Supplementary Information

Supplementary Table S1.

Supplementary Table S2.

Supplementary Table S3.

Supplementary Table S4.

## Data Availability

All data related to this manuscript are provided in the submission files.
